# The role of stem cell therapy in regeneration of dentine-pulp complex: a systematic review

**DOI:** 10.1007/s40204-018-0100-7

**Published:** 2018-09-28

**Authors:** Hengameh Bakhtiar, Amir Mazidi S, Saeed Mohammadi Asl, M. R. Ellini, A. Moshiri, M. H. Nekoofar, P. M. H. Dummer

**Affiliations:** 1 Endodontic Department, Dental Material Research Center, Tehran Dental Branch, Islamic Azad University, Tehran, Iran; 2 Stem Cell Research Center, Central Tehran Branch, Islamic Azad University, No. 4, 10th Neyestan St., Pasdaran Ave, Tehran, Iran; 3 Student Research Committee, Islamic Azad University, Tehran branch, No. 4, 10th Neyestan St., Pasdaran Ave, Tehran, Iran; 4 Department of Orthopedic Surgery, School of Medicine, AJA University of Medical Science, West Fatemi St, Etemadzadeh St, Tehran, Iran; 5 Department of Endodontics, School of Dentistry, Tehran University of Medical Sciences, North Karegar St, P.O. Box: 14395-433, Tehran, 14399-55991 Iran; 6 Endodontology Research Group, School of Dentistry, College of Biomedical and Life Sciences, Cardiff University, Heath Park, Cardiff, CF14 4XY UK

**Keywords:** Stem cell therapy, Dentin-pulp complex, Dentinogenesis, Tissue engineering, Regenerative medicine

## Abstract

Infection of the dental pulp will result in inflammation and eventually tissue necrosis which is treated conventionally by pulpectomy and root canal treatment. Advances in regenerative medicine and tissue engineering along with the introduction of new sources of stem cells have led to the possibility of pulp tissue regeneration. This systematic review analyzes animal studies published since 2010 to determine the ability of stem cell therapy to regenerate the dentine-pulp complex (DPC) and the success of clinical protocols. In vitro and human clinical studies are excluded and only the experimental studies on animal models were included. Dental pulp stem cells constitute the most commonly used cell type. The majority of stem cells are incorporated into various types of scaffold and implanted into root canals. Some of the studies combine growth factors with stem cells in an attempt to improve the outcome. Studies of ectopic transplantation using small animal models are simple and non-systematic evaluation techniques. Stem cell concentrations have not been so far reported; therefore, the translational value of such animal studies remains questionable. Though all types of stem cells appear capable of regenerating a dentine-pulp complex, still several factors have been considered in selecting the cell type. Co-administrative factors are essential for inducing the systemic migration of stem cells, and their vascularization and differentiation into odontoblast-like cells. Scaffolds provide a biodegradable structure able to control the release of growth factors. To identify problems and reduce costs, novel strategies should be initially tested in subcutaneous or renal capsule implantation followed by root canal models to confirm results.

## Introduction

As a consequence of caries, periodontal disease, trauma and several iatrogenic factors (Aksel and Serper 2014; Nagaveni et al. 2015), the human dentine-pulp complex (DPC) can develop reversible pulpitis that will progress to irreversible pulpitis and pulp necrosis without treatment. Currently, when the pulp is not savable, root canal treatment (RCT) is advised (Gong et al. 2016). Although RCT may be a successful option, the treated root canal system can become re-infected and/or the root may become susceptible to fracture (Li et al. 2016) leading either to the loss of the tooth or expensive and complex alternative treatments such as root canal retreatment, surgery, implants, etc.

Regenerative endodontics (RE) is a relatively new component of tissue engineering and regenerative medicine, which aims to introduce alternative options to classical treatment strategies (Na et al. 2013). The goal in RE is to replace the necrotic pulp with scaffolds, healing promoting factors, and cell therapies with the aim of regenerating new pulp and dentine within the root canal system (Galler et al. 2011). Different innovative methods including platelet concentrates (Bakhtiar et al. 2017a; Fakhr Tabatabayi et al. 2015) and treated dentine matrix (Bakhtiar et al. 2017b) products and stem cells have been used for this purpose. In this respect cell therapy is an important part of RE (Brar and Toor 2012; Rodriguez-Lozano et al. 2012) and various forms of stem cells may be utilized in order to provide the cells necessary for the regeneration of both the dentine and pulp (Alongi et al. 2010; Cao et al. 2015). The transplanted cells should be differentiated into various lineages including fibroblast, nerve cells, endothelial cells and odontoblasts to form new connective tissue, nerve fibers, blood vessels, and dentine (Shi et al. 2005). Thus, regeneration of the dentine-pulp complex is a complicated process (Schmalz and Smith 2014).

Because fully differentiated cells are aged and have low viability and limited matrix production soon after transplantation (Stolzing et al. 2008), stem cells are used as an alternative (Murakami et al. 2015) (Tables 1, 2, 3, 4 and 5). Stem cells have a number of advantages compared with differentiated cells (Murray et al. 2007), for example, they preserve their self-renewal capacity after transplantation and can be induced to differentiate into various cells lineages to be useful for regeneration of the dentine-pulp complex (Eslaminejad et al. 2007). Stem cells are classified into embryonic stem cells (Hilkens et al.), induced Pluripotent stem cells (iPSC) and adult/postnatal stem cells (Nuti et al. 2016). IPSCs and ESCs have received limited attention because of technical difficulties, ethical concerns and greater risk of carcinogenesis (Murray et al. 2007). Thus, studies on stem cells have concentrated on adult mesenchymal stem cells (Devolder 2010). Even though adult/postnatal stem cells have lower capacity for differentiation compared with ESCs and iPSCs, they can be used as a source of autologous grafting during an entire life (Casagrande et al. 2011).

**Table 1:** Variations of extracted data from reviewed articles

Endpoints	Variations (%)									
Cell type	DPSCs (52.2%)	SCAP (11.6%)	DFSC (8.7%)	BMSC (5.8%)	SHED (4.3%)	PDLSC (2.9%)	ADSC (2.9%)	Other (11.6%)	NA	NA
Scaffold	Collagen (25%)	TDM (13.3%)	HA/TCP (13.3%)	PLLA (10%)	PLGA (6.7%)	Atelocollagen (6.7%)	Fibrin gel (3.3%)	CBB (3.3%)	Silk fibroin (3.3%)	Other (15%)
Growth factors	TDM (18.9%)	BMP (16.2%)	G-CSF (16.2%)	SDF-1 (8.1%)	VEGF (8.1%)	b-FGF (5.4%)	FGF-2 (2.7%)	Other (24.3%)	NA	NA
Transplantation site	Subcutaneous (65%)	Inter canal (16.7%)	Renal capsule (13.3%)	Into socket (5%)	NA	NA	NA	NA	NA	NA
Animals	Mice (72.4%)	Rat (10.3%)	Swine (5.2%)	Dog (10.3%)	Rabbit (1.7%)	NA	NA	NA	NA	NA

*DPSCs* dental pulp stem cells, *PLLA* poly L-lactic acid, *PDLSC* periodontal ligament stem cell, *TCP* tricalciumphosphate, *HA* hydroxyl apatite, G-*CSF* granulocyte-colony stimulating factor, *BMSC* bone marrow stem cell, *SDF-1* stromal cell-derived factor-1, *DFSC* dental follicle stem cell, *APES* aligned PLGA/Gelatin electro spunsheet, *TDM* treated dentin matrix, *DPEM* dental pulp extracellular matrix, *PLGA* polylactic co glycolic acid. *ADSC* adipose-derived stem cell, *BMP* bone morphogenic protein, *PDLSC* periodontal ligament stem cell, *DPSCs* dental pulp stem cells. *TCP* tricalcium phosphate. *PLGA* polylactic co glycolic acid, *ADSC* adipose-derived stem cell. BMP: bone morphogenic protein, *SCAP* stem cell of apical papilla, *PDGF* platelet derived growth factor, *VEGF* vascular endothelial growth factor, *bFGF* basic fibroblast growth factor, *FGF* fibroblast growth factor

**Table 2:** Studies that transplanted stem cells into renal capsule

Reference	Animal model	Cell Type	Dose & dosage	Route of administration	Co-administrative factors	TERM approach	Time point	Main results
Hashmi et al. (2017)	Mice	BMSC	1 × 106 cells	Allograft Transplantation intorenal capsules	NA	Lyophilized hydrogel	2 week	Local mineralization Production of dentin-like tissue
Lei et al. (2013)	Rat		5 × 106 cells	Allogenic Dentin slice Transplantation intorenal capsule	Dentin slice	Dentin slices	6 weeks	Polarized cells penetrating into dentin wall
Yan et al. (2014)	Rat	SCAP	1 × 106 cells	Xenograft Transplantation intorenalcapsules	NA	SCAP pellets/root segment	8 weeks	MTA regulates dentinogenesis of SCAPs
Wang et al. (2013)	Rat		1 × 106 cells	Xenograft root fragment transplantation intorenal capsule	Different concentration of KH2PO4 (M2 > M1)	Root segments containing SCAP pellets/AGS	2 weeks	More mineralized tissues generation &, higher osteo/odontoblast differentiation in supplemented khpo4 medium
Wang et al. (2013)	Rat	DPSC	Not declared	Allogenic transplantation intorenal capsule	NA	AGS	2 weeks	Inflamed DPSC has more tendency to osteogenesis rather than dentinogenesis
Zheng et al. (2011)	Mice		5 × 106 cells/ml	Transplantation intosubrenal capsule	NA	PLLA/(HA,TCP & CDHA)	4 or 5 weeks	PLLA/TCP superiority for tooth tissue regeneration
Lei et al. (2011)	Rat	RPSC	106 cells each	allogeneic transplantation into the renalcapsules	NA	AGS	2 week	Typical dentinogenesis by iRPSC, bone-like tissues by mRPSC
Jiang et al. (2014)	Mice	Mesenchymal	2 × 105 cells	Rat to mice Transplantation intorenal capsule	hBMP4 hBMP7	PLGA	8 weeks	Enamel and dentin-like tissues generation in two integrated layers with amelogenin expression and amelo blastin

*BMSC* bone marrow stem cell, *NA* not assigned, *SCAP*: stem cell from apical papilla, *MTA* mineral trioxide aggregate, *hBMP* human bone morphogenic protein, *PLGA* polylactic co glycolic acid, *DPSC* dental pulp stem cell, *AGS* absorbable gelatin sponge, *BMSC* bone marrow mesenchymal stem cell, *PLLA* poly L-lactic acid, *HA* hydroxyl apatite, *TCP* tricalciumphosphate, *CDHA* calcium deficient hydroxyl apatite, *RPSC* root pulp stem cell, *TERM* Tissue engineering and regenerative medicine

**Table 3:** Models using subcutaneous transplantation

Reference	Animal Models	Cell type	Dose and dosage	Route of administration	Co-administrative factors	TERM approach	Time point	Main results
Zhang et al. (2015)	Mice	BMSC	1 × 10^7^ cells/ml	Xenograft subcutaneous cell-transplantation	SDF-1	Collagen	3 weeks	Participation of systemic BMSC in intracanal dental-pulp-like tissue regeneration
Yadlapati et al. (2017)	Mice	SCAP	2 × 10^4^ cells/well	Subcutaneous	VEGF	Poly dioxanone fiber	1.5–3 weeks	Blood vessel formation Negligible inflammation
Jin and Choung (2016)	Mice		1 × 10^7^ cell/100 mg powder	Xenograft Subcutaneous transplantation	rhPAI-1	HA/TCP ceramic powder fibrin gel	12 weeks	Dentin formation Odontoblast presses inserted to dental tubules
Wang et al. (2016)	Mice		3 × 106 cells and 1.5 × 105 NF-MS (20:1 ratio) mixed	Xenograft Subcutaneous injection	BMP-2	PLLA NF-MS + PLGA MS	4, 8 weeks	Mineralized tissue with embedded cells resembling osteodentin excellent microenvironment for SCAP to regenerate dentin tissue
Na et al. (2013)	Mice		2 × 10^6^ cells	Xenograft subcutaneous implantation	hTDM	TDM	6 weeks	SCAP-CSDPs with amount of endogenous ECM capable of forming a heterotopic dental pulp/dentin complex
Huang et al. (2010)	Mice		107 cells/mL	Xenograft Subcutaneous root fragment transplantation	NA	PLG	21–28 weeks	Fulfilling vascularization, continuous dentin-like tissue deposition
Hilkens et al. (2017)	Mice		NR	Xenograft Subcutaneous transplantation	NA	HA/TCP hydrogel	8, 12 weeks	Function of vascularized pulp-like tissue
Zhang et al. (2017)	Mice	DPSC	NR	Xenograft subcutaneous transplantation	PDGF	Calcium phosphate cement	12 week	Facilitated cell growing and more mineralized dentin like tissue by PDGF
Kawamura et al. (2016)	Mice		1 × 10^4^ cell	Xenograft Subcutaneous transplantation	EDTA-treated dentin slice	Collagen TE	4 weeks	Lower pulp-dentin regeneration compared with HCL treated dentin slice
Atalayin et al. (2016)	Mice		NR	Subcutaneous	NA	HA/TCP PLDL PDL	6,12 weeks	Highest expression of DSPP in PDL, DMP-1 in HA/TCP
Li et al. (2016)	Mice		5.0 × 10^6^ cells/mL	Xenograft Subcutaneous tooth transplantation	VEGF	PLLA HG-MS	9 weeks	Full-length high blood vessel apically maximum pulp-like tissues no effect of VEGF on proliferation of DPSC.
Tran Hle and Doan (2015)	Mice		3 × 10^4^ cells/scaffold	Xenograft subcutaneous transplantation	hTD	hTD	4,6,8 weeks	Dentin-like tissue formation with itse special markers
Yang et al. (2015)	Mice		5 mL of cells (10^7^/mL) per scaffold	Xenograft subcutaneous tooth fragment	b-FGF	Silk fibroin	7 weeks	Generation of Pulp-like tissue
Dissanayaka et al. (2015)	Mice		1 × 10^6^ cells/mL	Xenograft subcutaneous root fragment	NA	Peptide nano fibrous (peptide hydrogel	4 weeks	Vascularized pulp-like tissue with patches of osteodentin
Takeuchi et al. (2015)	Mice		5 × 10^5^ cells	Xenograft subcutaneous transplantation	b-FGF G-CSF	Collagen TE	3 weeks	No difference between bFGF and G-CSF in the regeneration
Horibe et al. (2014)	Mice	DPSC	1 × 10^6^ cells	Xenograft subcutaneous tooth slice	NA	collagen TE	3,4 weeks	Similar regeneration of MDPSCs from young and aged donors
Dissanayaka et al. (2014)	Mice		256,000/190 µL	Xenograft subcutaneous tooth slice	NA	3D microtissue spheroids	4 weeks	Vascular and pulp like tissue regeneration
Wang (2014)	Mice		1 × 10^6^ cells	Xenograft subcutaneous transplantation	NOV???(nephroblastoma overexpressed)	Porous PLGA	2, 3, 4 weeks	Promotion of dentinogenesis and odontoblastic differentiation
Lei et al. (2014)	Mice		5 × 103 cells/well	Xenograft subcutaneous transplantation	NA	NA	8 weeks	Maintained MSC characteristics after implantation (DPSC > PDLSC)
Yang et al. (2012)	Mice		1.0 × 10^6^ cells/mL	Xenograft subcutaneous implantation	Plasmid vectors encoding BMP-7	Chitosan/collagen	4 weeks	Odontoblast-like phenotype differentiation
Wang et al. (2012)	Mice		2.0 × 10^6^ cells	Xenograft subcutaneous implantation	NA	Fibrin gel CBB	8 weeks	Capability of mineralization SHEDs > DPSCs CT formation SHEDs < DPSCs
Chen et al. (2012)	Mice		3 × 10^6^ cells/mL	Xenogenic subcutaneous transplantation	NA	HA/TCP Cell sheets/powdery HA-TCP scaffolds/root fragments	12 weeks	PL enhances the and layer of odontoblast-like cell formation
Wang et al. (2011b)	Mice		5 × 10^6^ cells	Xenograft subcutaneous transplantation	NA	CBB particles	6 weeks	Regular dentin–pulp complex and columnar odontoblast-like cells generation
Wang et al.(2011a)	Mice	DPSC	10^6^ cells	Xenograft subcutaneous transplantation	NA	NF-PLLA	8 weeks	Enhanced odontogenic differentiation of human DPSCs and mineralization in NF-PLLA
Lee (2011)	Mice		1 × 10^7^ cells	Xenograft subcutaneous transplantation	pre ameloblast-CM	HA/TCP ceramic powder	6.12 weeks	Dentin deposition with palisaded odontoblast-like cells formation
Galler et al. (2011)	Mice		1 × 10^6^ cells/100µL	Xenograft subcutaneous in dentin cylinder transplantation	VEGF, TGFb1, FGF2	Laden peptide hydrogel	6 weeks	Generation of odontoblasts-like phenotypes, vascularization
Alongi et al. (2010)	Mice		4 × 10^6^ cells	Xenograft subcutaneous transplantation	NA	HA and TCP powder	8 weeks	DPSCs from inflamed pulp formed pulp/dentin complexes in lesser extent than DPSCs–NPs
Wang (2010)	Mice		1 × 10^6^ cells	Xenograft subcutaneous transplantation	BMP-7 + dexamethasone	NF-PLLA	8 weeks	More organized odontoblast like cells formation
Ma et al. (2012)	Mice	SHED	4 × 10^6^ cells	Xenogenic subcutaneous transplantation	NA	HA/TCP	8 weeks	Mineralization & DPC generation equally in SHED Fresh and SHED-Cryo
Wang et al. (2012)	Mice		2.0 × 10^6^ cells	Xenograft subcutaneous implantation	NA	Fibrin gel CBB	8 weeks	Capability of mineralization SHEDs > DPSCs CT formation SHEDs < DPSCs
Jeon et al.(2014)	Mice		3 × 10^6^ cells	Xenograft Subcutaneous transplantation	NA	macroporous biphasic calcium phosphate	9 weeks	Hard tissue formation (o-SHED > e-SHED) quality of hard tissue (o-SHED = e-SHED)
Chen et al (2015b)	Mice	DPC/DFC	1 × 10^7^ cells/mL	Allograft subcutaneous transplantation	TDM	TDM	6 weeks	Similar dentin-like tissue formation
Tian (2015)	Mice		5 × 104 Cells/scaffold	Xenograft subcutaneous transplantation	TDM	TDM	8 Weeks	The structure of dentin tissues generated by DFCs was more complete
Jiao et al. (2014)	Mice		1 × 10^4^ cells	Xenograft subcutaneous transplantation	dentin matrix	Human TDM and CDM	8 weeks	More mechanical properties dentinogenic protein release by CDM
Guo (2013)	Mice		5 × 10^4^ cells in each	Xenograft subcutaneous implantation	TDM	TDM	8 weeks	Formation of pulp-dentin/cementum/periodontium-like tissues
Yang et al. (2012)	Mice		1 × 106 cells/mL	Xenograft subcutaneous implantation	TDM	TDM	8 weeks	New dentin-pulp like tissues and cementum-periodontal complexes
Lei et al. (2014)	Mice	PDLSC	3 × 104 cells/dish	Xenograft subcutaneous transplantation	NA	NA	8 weeks	Maintained MSC characteristics after implantation (DPSC > PDLSC)
Tian (2015)	Mice		5 × 10^4^ Cells/scaffold	Xenograft subcutaneous transplantation	TDM	TDM	8 weeks	The structure of dentin tissues generated by DFCswas more complete
Chen et al. (2015c)	Mice	UCMSC	5 × 10^4^ cells/well	Xenograft subcutaneous transplantation	hTDM	TDM	8 weeks	Formation of layers of cells and calcifications
Ishizaka R (2013)	Mice	Pulp CD31- (SP)	1 × 10^6^ cells in each	Xenograft subcutaneous implantation	NA	Collagen TE	4 weeks	Angiogenesis, neurogenesis and pulp regeneration induction
Pan et al. (2013)	Mice	Human DP progenitors	10^6^ cells/50 μl	Xenograft subcutaneous implantation	Stem cell factor (SCF)	Collagen sponge	4 weeks	Induction of cell homing, angiogenesis, and tissue remodeling
Huo et al. (2010)	Mice	Dermal multi potent cells	2.0 × 10^6^ cells	Xenograft subcutaneous transplantation	Embryonic and neonatal TGC-CM	Fibrin gel	4 weeks	Bone like structure formation from embryonic TGC-CM

*1-PDGF* platelet derived growth factor, *2-DPSC* dental pulp stem cell, *3-SCAP* stem cell of apical papilla, *4-NA* not assigned, *5-HA* hydroxyl apatite, *6-TCP* tricalcium phosphate, *7-VEGF* vascular endothelial growth factor, *8-EDTA* ethylenediaminetetraacetic acid, *9-HCL* hydrochloric acid *10-rhPAI-1*, *11-PLDL* copolymer of L-lactide and DL-lactide, *12-PDL* copolymer of DL-lactide, *13-DSPP* dentin sialo-phosphoprotein, *14-DMP-1* dentin matrix protein-1, *15-PLLA* poly L-lactic acid, *16-HG* heparin-conjugated gelatin, *17-MS* microsphere, *18-BMP* bone morphogenic protein, 19-NF: nanofibrous, *20-PLGA* poly lactic co glycolic acid, *21-hTD* human treated dentin, *22-BMSC* bone marrow stem cell, *23-SDF-1* stromal cell-derived factor1, *24-bFGF* basic fibroblast growth factor, *25-UCMSC* umbilical cord mesenchymal stem cell, *26-hTDM* human treated dentin matrix, *27-DFC* dental follicle cell, *28-DPC* dental pulp cell, *29-CNCC* cranial neural crest cell, *30-PDLSC* periodontal ligament deciduous teeth, *31-NOV* nephroblastoma overexpressed, *32-CDM* cryopreserved dentin matrix, *33-CBB* ceramic bovine bone, *34-CM* condition medium, *35-TGF* transforming growth factor, *36-FGF* fibroblast growth factor, *37-TGC* tooth germ cell, *38-PLG* poly lactide and glycolide, *TERM* Tissue engineering and regenerative medicine

**Table 4:** Models that transplanted stem cells into the jawbone or extracted socket

Reference	Animal model	Cell type		Dose & dosage	Route of administration	Co-administrative factors	TERM approach	Time point	Main results
Gao et al. (2016)	Pig	PDLSC		2 × 10^5^ cells	Allogeneic direct implantation into socket	Vitamin C	PDLSC sheet + HA/ TCP/DPSCs	24 weeks	Generation pfbio-root with normal pulp and dentin-like matrix and natural biomechanical structure in low rate.
		DPSC		2 × 10^6^ cells					
Kodonas et al. (2012)	Pig	DPSC		3 × 10^6^ cells	Autologous root fragment transplantation into jawbone	NA	Collagen PLGA	6-10 weeks	Formation of continuous polarized & non-polarized cell along the canal wall
Hung et al. (2011)	Rabbit	ADSC	DPSC	5 × 10^6^ cells/ml	Autologous transplantation into the extracted socket	BMP-2	Collagen gel	12 weeks	Similar tooth structure by different stem cells close to a normal living tooth

*1-PDLSC* periodontal ligament stem cell, *2-DPSC* dental pulp stem cell*. 3-HA* hydroxyapatite, *4-TCP*: tricalcium phosphate. *5-PLGA* polylactic co glycolic acid, *6-ADSC* adipose-derived stem cell, *7-BMP* bone morphogenic protein, *TERM* Tissue engineering and regenerative medicine

**Table 5:** Models that transplanted stem cells into root canal

References	Animal model	Cell type	Dose and dosage	Route of administration	Co-administrative factors	TERM approach	Time point	Main results
Kuang et al. (2016)	Rat	DPSC	8 × 10^6^ cells + 5 × 10^5^ microsphere e + 2 ml culture	Xenograft intracanal transplantation	Hypoxic treatment	PLLA Nanofibrous spongy microsphere	4 weeks	Enhanced vascularization
Iohara et al.(2016)	Dog		1 × 10^4^ cell/cm^2^	Autologous interacanal transplantation	G-CSF	Atelocollagen	13–26 weeks	Normal pulp-like tissue and apical secondary dentin formation
Nakashima M (2014)	Dog		2 × 104 cells/100 mL	Autologous interacanal transplantation	G-CSF	Drug approved collagen	2, 4, 9, 26 weeks	Regeneration of vascularized pulp tissue, dentin deposition along dentin wall and dense nerve plexus
Iohara et al. (2014)	Dog		1 × 106 cells/ml	Autologous interacanal transplantation	G-CSF	Atelocollagen	2,17 weeks	Pulp-like tissue regeneration 60% apically, dentin & nerve formation
Iohara et al. (2013)	Dog		1 × 10^6^ cell per ml	Autologous intracanal transplantation	G-CSF	Clinical-graded atelocollagen	2, 4, 9, 26 weeks	Over 90% pulp-like tissue regeneration, dentin & dense nerve plexus formation
Wang et al. (2013)	Beagles		2.0 × 10^7^ cells	Autologous transplantation into the pulp canal	NA	Gel foam	24 Week	Generation of pulp-like tissues
Murakami et al. (2015)	Dog	BMSC	5*10^5^ cells	Autologous interacanal transplantation	G-CSF	Atelocollagen	2 weeks	Potential pulp regeneration in MADSC & MBMSC but in less volume
Yang et al. (2015)	Beagles	NR	NA	Cell homing	SDF-1α	Silk fibroin	12 weeks	Pulp tissue generation and mineralization along dentinal wall
Iohara et al. (2011)	Dog	Pulp CD 10^5^ + cells Adipose CD 10^5^ + cells	1 × 10^6^ cells in each	Autologous intracanal transplantation	SDF-1	Collagen TE	2, 4, 13 week	Full length pulp-like tissue formation, odontoblastic lining & tubular dentin along dental wall
Chen et al. (2015a)	Pig	DFSC	1 × 10^6^ cells	Direct implantation into socket	APES/TDM/DPEM	APES/TDM/DPEM	12 weeks	Generation of uniform pulp-like tissue, predentin matrix formation

*1-DPSC* dental pulp stem cell, *2-PLLA* poly L-lactic acid, *3-PDLSC* periodontal ligament stem cell, *3-TCP* tricalcium phosphate, *4-HA* hydroxyl apatite, *5-G-CSF* granulocyte-colony stimulating factor, *6-BMSC* bone marrow stem cell, *7-NA* not assigned, *8-SDF-1* stromal cell-derived factor-1, *9-DFSC* dental follicle stem cell, *10-APES* aligned PLGA/Gelatin electrospun sheet, *11-TDM* treated dentin matrix. *12-DPEM* dental pulp extracellular matrix, *13-PLGA* polylactic co glycolic acid, *14-ADSC* adipose-derived stem cell, *15-BMP* bone morphogenic protein, *TERM* tissue engineering and regenerative medicine

Many sources of adult/postnatal stem cells have been investigated in the mouth including the dental pulp, periodontal ligament (PDL), dental follicle, gingiva, bone, alveolar bone, and papilla (Egusa et al. 2012). Among these, dental pulp stem cells (DPSCs) are easiest to access; they also have a greater differentiation capacity and are widely used in dental research (Nuti et al. 2016). Furthermore, adult/postnatal stem cells can remain undifferentiated when they are not exposed to differentiating signaling molecules (Schmalz and Smith 2014), are capable of long term self-replication, and maintain their capacity for multiple differentiation during the entire life of organs (Barry 2003).

The developing science of stem cells has succeeded in regenerating parts of the heart (Chong and Murry 2014), muscles (Dellavalle et al. 2011), bone (Asatrian et al. 2015) and the nervous system (Reynolds and Weiss 1992). Such developments have also included oral and dental tissues (Ikeda et al. 2009). For example, stem cells have been used for regeneration of the periodontium, alveolar bone, dentine-pulp complex, craniofacial bone, mucosal tissue, tongue muscle, and for returning the function of salivary glands (Liu and Cao 2010; Rimondini and Mele 2009). Because of complications with whole tooth regeneration, substantial efforts have been made to regenerate the dentine-pulp complex (Gao et al. 2016). Although adult/postnatal stem cells therapy has been the focus of many studies, a lack of consensus on the actual efficacy of adult/postnatal stem cells for dentine-pulp regeneration, has constrained its clinical value.

This study was designed to evaluate in vivo animal studies that have used adult/postnatal stem cells to determine whether adult/postnatal stem cells therapy is able to regenerate new dentine-pulp complexes and which of the many available protocols can be translated into the clinical setting. Therefore, laboratory and human clinical studies were excluded.

## Review

### Search strategy

**Fig. 1:**
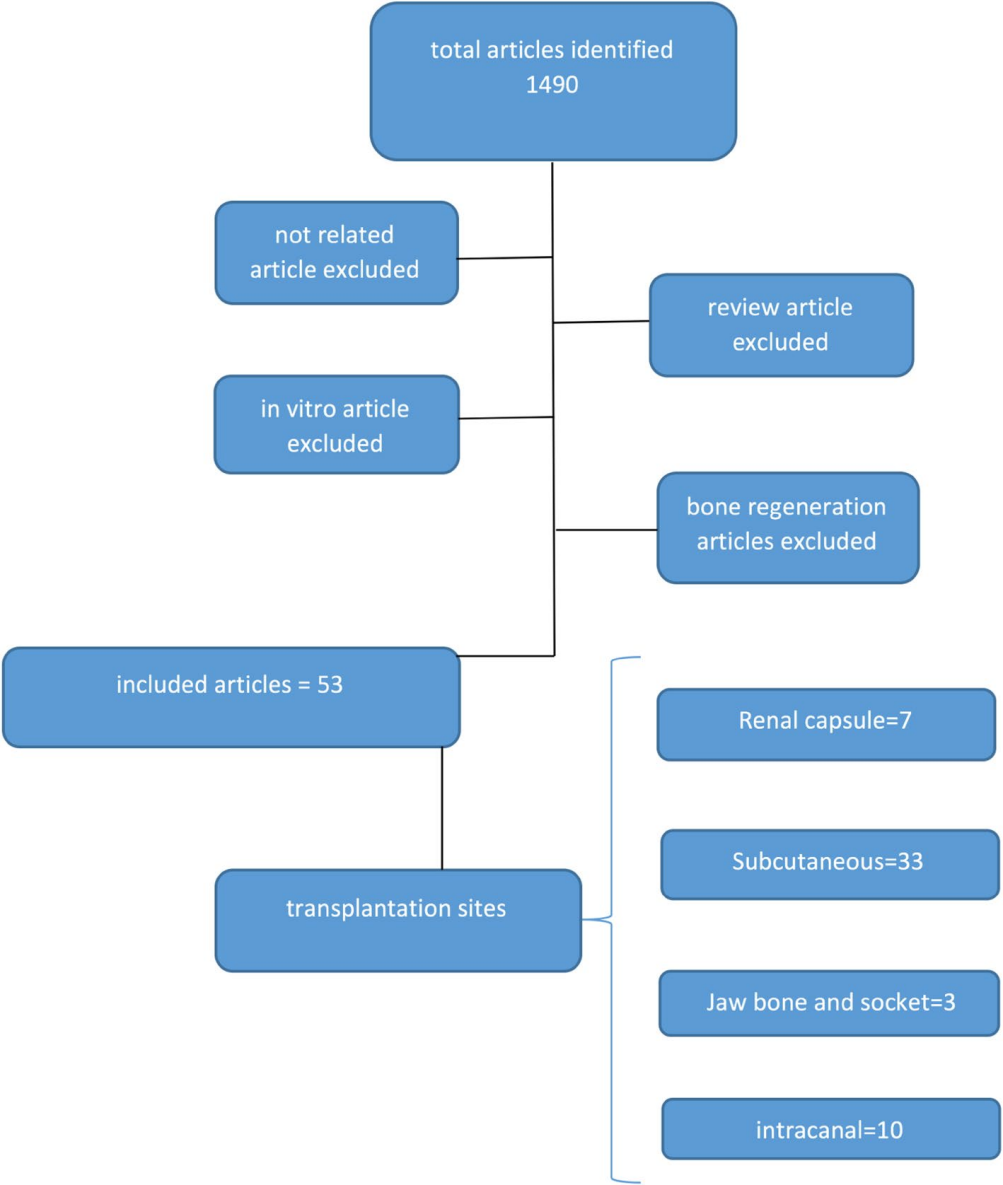
Search strategy

English scientific reports published since 2010 and indexed in PubMed were searched. The main key terms included: “stem cell”, “dentin-pulp complex”, “dentinogenesis” and “pulp regeneration”. Because regeneration of the dentine-pulp complex must be evaluated in vivo and since most of the studies are performed in animal models, the in vitro and human clinical studies were excluded and only experimental studies on animal models were included in the present review. Specific aspects of the studies including animal models, type of stem cells used for pulp regeneration and their sources, concentration of the administered cells, route of administration, co-administrative factors, tissue engineering approaches of the cell therapy, time point evaluation of the regeneration process and the main result of each study were reviewed and evaluated to allow comparisons. Overall, 1490 articles which were identified in our search included; unrelated articles, in vitro cases, studies with bone regeneration approaches; by excluding review articles; and finally, 60 studies that focused on the role of adult/postnatal stem cell therapies for regeneration of the dentine-pulp complex in animal models were included (Fig. 1).

### Types of stem cells

All the stem cells used for dentine-pulp regeneration were adult mesenchymal stem cells (MSCs); however, the source of harvesting the MSCs varied in different studies. Dental pulp stem cells (DPSCs) were the first dental stem cells to be isolated and their odontogenic, neurogenic, and angiogenic properties were reported in several studies (Dissanayaka et al. 2015; Kuang et al. 2016; Wang et al. 2013). These cells were capable of being harvested during the early stages of life and were stored for future use (Tran Hle and Doan 2015). The majority (52%) of the studies used DPSCs for regeneration of dentine-pulp complexes (Tables 1, 2, 3, 4 and 5).

Among the 60 studies reviewed 36 cases used DPSCs (Tables 2, 3, 4 and 5). Of the 8 studies that transplanted stem cells into the renal capsule, 2 cases used DPSCs (Table 2). In 39 studies of subcutaneous transplantation, 25 of them used DPSCs (Table 3). Of the 10 studies with intracanal transplantation, seven used DPSCs (Table 5). Of the 60 studies that were reviewed, 8 used SCAPs (Table 1, 2, 3, 4 and 5). In two studies the cells were transplanted into the renal capsule (Table 2) and six of the studies transplanted the cells subcutaneously (Table 3). No studies used SCAPs transplanted into root canals (Table 5). In the evaluated studies, 2 studies attempted to regenerate PDL tissue by means of PDLSCs, one transplanted subcutaneously and one transplanted into an extraction socket (Tables 3 and 4). In total 3 studies used SHEDs and all used a subcutaneous approach (Table 3). Three studies used BMSC, 2 in the renal capsule, 1 subcutaneously and the other was transplanted in a root canal (Tables 2, 3 and 5). Only Chen et al. (2015c) transplanted UCMSC subcutaneously (Table 3). Of the 60 studies evaluated, 9 used autologous stem cells (Tables 2, 3, 4 and 5). None of the studies with a renal or subcutaneous transplantation approach used autologous stem cells (Table 2 and 3). One subcutaneous transplantation case and one renal capsule model used allograft stem cells and the rest (47 out of 49 studies) used xenograft stem cells (Table 3).

### Dental pulp stem cells

In all studies using DPSCs, they isolated the stem cells from human healthy pulp tissue to be used in their animal model, usually from orthodontically extracted teeth, for instance third molars were often used (Chen et al. 2012). Alongi et al. (2010) reported that inflamed pulp tissue was an appropriate source for isolation of DPSCs. In their study inflamed pulp-derived stem cells revealed a capacity for regeneration of the dentine-pulp complex, albeit the regeneration was weaker compared with the control group where the cells were derived from intact pulps (Alongi et al. 2010). It has also been reported that stem cells from an exposed pulp are more prone to differentiate into osteoblastic cells rather than dentinogenic cells (Wang et al. 2013).

### Stem cells from apical papilla

As an element of a developing tooth, the stem cells of the apical papilla (SCAP) have a greater stem capacity (Huang et al. 2010; Wang et al. 2016). Stem cells of the apical papilla are known for more rapid proliferation and mineralization, better migration and telomerase activity than DPSCs (Huang et al. 2010). Wang et al. (2016) reported deposition of more uniform dentine-like tissue created by SCAPs than DPSCs with greater similarities to natural dentine. Stem cells of the apical papilla were commonly isolated from immature third molars.

### Periodontal ligament stem cells

Periodontal ligament stem cells (PDLSCs) have been used to create PDL (periodontal ligament) in studies attempting to regenerate a new bio-root (Gao et al. 2016). They achieved a bio-root with a suitable PDL tissue using a combination of DPSCs and hydroxyapatite, which were wrapped by a sheet of PDLSCs. These newly generated roots in miniature pigs, had similar qualities to natural teeth in both mineral component and biomechanical properties but successful results were achieved in only one-fifth of the samples while titanium implants were 100% successful (Gao et al. 2016).

### Stem cells from human exfoliated deciduous teeth

Stem cells from human exfoliated deciduous teeth (SHED) are another type of stem cell, which are derived from extracted deciduous teeth and are considered as a non-invasive source of stem cells (Jeon et al. 2014). These stem cells have an enhanced capacity for osteogenic regeneration and higher proliferation rate compared with DPSCs (Wang et al. 2012).

### Bone marrow derived mesenchymal stem cells

Bone marrow derived mesenchymal stem cells (BM-MSCs) are another source that has been used extensively in regenerative procedures (Lei et al. 2013). Use of such cells with a dentine matrix scaffold was associated with differentiation of the stem cells into polarized odontoblast-like cells with penetrating processes into dentinal tubules (Lei et al. 2013). However, harvesting these cells from human sources is an invasive procedure and its main clinical application is in orthopedic research (Chen et al. 2015d). Meanwhile, Zhang et al. (2015) suggested the use of endogenous BM-MSC for regenerating lost tissue after observing its systemic homing to the root canal, powered by application of stromal cell-derived factor-1 (SDF-1), in a subcutaneously transplanted tooth with a root canal.

### Adipose-derived stem cells

Hung et al. (2011) used adipose-derived stem cells (ADSCs) due to their large population in mammals and higher rate of proliferation with similar results to DPSCs in tooth regeneration. While harvesting DPSCs is achieved primarily from the healthy pulp of a tooth, use of ADSCs could be more convenient. Murakami et al. (2015) reported that despite the superiority of DPSCs, sufficient ADSCs and bone marrow derived mesenchymal stem cells could be considered as an alternative to DPSCs.

### Umbilical cord mesenchymal stem cells

Umbilical cord mesenchymal stem cells (UCMSC) are available in large volumes without invasive harvesting procedures and are stored in worldwide stem cell banks (Chen et al. 2015c). They reported UCMSC capacity for differentiation into odontoblast-like cells and deposition of hard tissue. Notably, these cells are considered safe as they are protected from viral infections by the placenta, which has a significant clinical importance (Chen et al. 2015c).

### Sources of stem cell

Although using autologous stem cell grafts are a priority and conforms to regulatory policies, there are limitations for harvesting autologous stem cells in elderly patients (Wei et al. 2013). This study reported a promising capacity for using allograft stem cells for tooth regeneration in their studies on miniature pigs for developing a bio-root.

Before administering stem cells for regeneration, they need to be cultured to achieve the required quantity, especially when considering human derived stem cell due to their limited numbers (Asatrian et al. 2015; Chen et al. 2012; Dissanayaka et al. 2014). Traditionally, cell culturing is undertaken with fetal bovine serum, which increases the risk of transinfection and immunologic responses (Chen et al. 2012). Researchers have used human platelet lysate for cell culturing during pulp tissue regeneration, implying that the use of autologous medium is a possibility (Chen et al. 2012). Besides increasing the quantity of the stem cells, culturing stem cells with different vehicles can induce them to differentiate into specific target tissues. For instance, dexamethasone and ascorbic acid in culture media lead to greater osteogenic differentiation (Wang et al. 2013). Tooth germ cell-condition medium (TGC-CM) has been introduced for its inductive properties in odontoblastic differentiation (Huo et al. 2010), which can be prepared from three sources: human, rat and porcine. Wang et al. (2011b) reported that culturing DPSCs using porcine-derived TGC-CM resulted in greater regular odontoblast-like cell layer formation compared with human-derived TGC-CM. Huo et al. (2010) prepared TGC-CM from rats in two stages, embryonic and neonatal, and cultured dermal multipotent stem cells in these two media. They observed that embryonic TGC-CM was more bone inductive rather than odontoblastic (Huo et al. 2010). In mineralization-inductive media, supplementary amounts of KH_2_PO_4_ can make the stem cells more potent for odontoblast or osteoblast differentiation (Wang et al. 2011a, 2013). Only these two studies have used this culture medium in their subcutaneous transplantation models (Table 3).

### Animal models

Rodents were the most widely used animals (Tables 2, 3, 4 and 5), probably due to their genetic similarities to humans. In addition, rodents are cheaper with a more rapid rate of birth that makes them suitable for in vivo studies. Among rodents, the mice and rat were the most popular animal models used in large numbers probably because of their low cost, which led to. Rabbit was another animal that was used, but it was less popular and used less frequently. Pigs and dogs were also used. Among the 60 studies evaluated, 44 studies used mice as the animal model. All the 39 studies (100%) involving a subcutaneous transplantation approach used mice (Table 3) whereas no study involving intracanal transplantation used mice except one study, which used rats (Table 5). From eight studies with transplantation into the renal capsule, three used mice and 5 used rats, thus mice and rats were the animals of choice for transplantation into the renal capsule (Table 2). Pig and rabbit were used as animal models in 3 studies where the transplantation was in the jawbone or the extraction socket (Table 4). Among 10 intracanal transplantation studies evaluated, all used dogs except for one (Kuang et al. 2016) that used rats.

### Route of administration

Most of the studies on tooth slices, containing a root canal with overlying dentine as implantation of complete root structures in rodents, are challenging. Slices were then subcutaneously implanted in the dorsum of animals and the cells were either seeded within a form of scaffold or hydrogel in the centre of the slices corresponding to the root canal (Alongi et al. 2010; Dissanayaka et al. 2014, 2015; Horibe et al. 2014; Huang et al. 2010; Ishizaka et al. 2013; Lei et al. 2014; Takeuchi et al. 2015; Tran Hle and Doan 2015; Yan et al. 2014; Yang et al. 2015b; Zhang et al. 2015). However, tooth slices had variable thickness, which significantly affected the reliability of the studies.

The best environment that simulates the real situation is a pulpectomized canal of a tooth in the alveolus of an animal (Table 4) since this simulates better the human situation. In this way, because all clinically relevant factors are included, outcomes are more generalizable and conclusive. As stated, however, orthograde regenerative endodontic procedures with stem cells have been performed in few studies on larger animal models such as dogs. Of the 60 studies evaluated in the present review, 47 (78%) used subcutaneous (65%) or a renal capsule (13%) implantation model in a retrograde manner while 10 studies (22%) used an orthograde model (true root canal model 17% and into jaw bone or socket, 5%).

### Tissue engineering and regenerative medicine approach (biomaterials)

Scaffolds have a major role during cell therapy. In fact, most of the in vivo studies that administered stem cells for regeneration of the dentine-pulp complex used a type of scaffold combined with stem cells. Beside delivery of stem cells, carriers (scaffolds) also act as carriers for growth factors to control their release (Wang et al. 2016; Yang et al. 2012). Tissue scaffolds vary widely; based on their structure and architecture, they may be fibrous or spongy with variable pore size and porosity (Kuang et al. 2016). Based on the material properties, scaffolds may be natural, synthetic or hybrid with variable drug delivery, cell behavior, in vivo behavior and biophysicochemical properties (Ajay Sharma et al. 2015; Hilkens et al. 2014; Tran Hle and Doan 2015).

In designing suitable scaffolds for dentine-pulp regeneration they should mimic the native environment of the dentine-pulp area to trigger stem cells to differentiate into various cell lineages (Ajay Sharma et al. 2015; Chen et al. 2015a; Dissanayaka et al. 2014). In addition, an optimum scaffold should be porous (Kuang et al. 2016). Thus, spongy scaffolds may be the superior option since their greater porosity allows stem cells to migrate, proliferate and attach to the scaffold sheet as well as encourage the stem cells to synthesize a homogenous matrix (Kuang et al. 2016). Thus, such porous scaffold should have good porosity with large diameter interconnecting pores (Nagaveni et al. 2015; Wang et al. 2011a). Porous structures such as nanofibrous microspheres also make fewer by products after degradation due to their lower density compared with non-porous structures such as solid microspheres (Li et al. 2016; Nagaveni et al. 2015; Wang et al. 2016). In addition, the scaffolds should be biocompatible and biodegradable in vivo (Sharma et al. 2014). An incompatible scaffold would trigger inflammation over a long period of time and a non-biodegradable or even slow biodegradable scaffold would retard new tissue ingrowth and prevent uniform matrix formation (Chan and Leong 2008).

A biomimetic scaffold for dentine-pulp regeneration should be a biphasic structure with a suitable medullary region for pulp regeneration and a cortical region suitable for dentine regeneration. Since the pulp area is chiefly composed of organic tissue the medullary element of such a biomimetic scaffold should be fabricated from organic materials such as gelatin, collagen, elastin, fibrin, etc. Because hydroxyapatite forms the greater percentage of dentine (Goldberg 2011), the outer cortical area of such a scaffold should be made-up of inorganic materials such as hydroxyapatite and tricalcium phosphate (Wang et al. 2013). To improve scaffold drug delivery and mechanical properties, small amounts of synthetic materials such as polylactic co glycolic acid (PLGA), polylactic acid (PLA), polycaprolactone (PCL) and polyglycolic acid (PGA) may be incorporated into the medullary and cortical regions of the desired scaffold as a basic mesh (Zheng et al. 2011). In addition, due to the irregular shape of pulp canals, injectable scaffolds with small particle sizes are desirable (Nagaveni et al. 2015).

After evaluating the 60 studies in the present review, of the 40 studies with transplantation into the renal capsule or subcutaneously, 8 used treated dentine matrix (TDM) and 10 used calcium/phosphate-containing compounds such as hydroxylapatite (HA) or tricalcium phosphate (TCP) as the carrier for stem cells whereas no study transplanted TDM into the renal capsule and only one study used calcium/phosphate-containing compounds for renal capsule transplantation (Tables 2 and 3). Among the 60 studies, 6 used PLLA, four used PLGA, 15 used collagens, 2 used fibrin, 2 used fibroin and 1 used gel foam (Tables 2, 3, 4 and 5). Of 8 studies using renal capsule transplantation, 3 used absorbable gelatin sponge (AGS) and there was no use of AGS in the other three approaches (Table 2). On the other hand, no collagen was used in the renal capsule approach, whereas it was popular in other approaches (Table 2). Only 2 studies out of 60 used hydrogels and those were transplanted subcutaneously (Table 3). Only 1 study used microtissue cells without a carrier in subcutaneous transplantation (Table 3). The transplantation was accompanied with a slice of root or dentine in 3 studies with renal capsule transplantations and 1 study with subcutaneous transplantation (Tables 2 and 3).

### Dose

Cell concentration is an important criterion when stem cell therapy is designed for dentine-pulp regeneration. There was a lack of significant strategy to estimate the dose of stem cells appropriate for dentine-pulp regeneration complex in these 60 studies. High doses of stem cells may have an inhibitory effect on regeneration as the nutrient supply of the pulp is restricted (Zheng et al. 2012). On the other hand, low doses of stem cells lead to less tissue generation. Further, scaffolds have a specific surface area for adhesion and their structure determines the amount of nutrient supply; therefore, specification of the dose of stem cells is directly related to scaffold design (Zheng et al. 2012). The manufacturer usually reports the optimal number of cells in commercially available scaffolds but in the in vivo research such numbers are estimated from previous in vitro research (Zheng et al. 2012). Based on the studies that were reviewed the main determining factor for the dose of stem cells seemed to be the laboratory procedure of cell seeding onto the scaffolds that varied widely in each study, thus drawing general recommendations is not possible. Overall, 41% of the evaluated studies reported a range of 10^6^–10^7^ stem cells (Tables 2, 3, 4 and 5).

### Co-treatment factors

Healing promotive factors include a wide variety of growth factors, drugs, bioactive materials, glycosaminoglycans and other small molecules and peptide motifs that may be used with stem cells and scaffolds to enhance the effectiveness of the stem cell therapy on dentine-pulp regeneration and scaffold biocompatibility and biodegradability. Growth factors have a short half-life so should be encapsulated in degradable materials to control their release (Li et al. 2016; Nagaveni et al. 2015). Of the 60 studies evaluated, 23 used no co-administrative factor in combination with stem cells (Tables 2, 3, 4 and 5). Almost half of the studies using a retrograde approach into renal capsule transplantation (63%) and subcutaneous transplantation (38%) had assigned no type of co-treatment factor (Tables 2 and 5), whereas in orthograde (regeneration along the full length of the root). approaches, among 13 studies, only 2 (15%) used nothing (Tables 4 and 5). Generally, treated dentine matrix (TDM) and its soluble proteins were the most popular co-treatment factor. Of 39 studies with a subcutaneous transplantation approach, seven (18%) used TDM (Table 3). In intracanal studies, these were not popular (only one study) probably because of the existence of natural dentine at the site (Table 5). Of 37 studies that applied co-treatment factors, 6 (16%) used BMPs, 6 used G-CSF (16%), 3 used SDF-1 (8.1%), three (8%) used bFGF, and 3 (8%) used VEGF (Tables 2, 3, 4 and 5). These percentages are based on the number of studies with the application of co-treatment factors. Due to the overlap of studies and combination use of co-treatment, the percentages in the pie chart are different, as these are based on the number of co-administrative factor types.

### Other related factors

The impact of age on the capacity of stem cells is a critical aspect of stem cell therapy. Iohara et al. (2014) reported that there was little difference in the regenerative potential of stem cells derived from old or young donors; however, in vivo experiments on canine models reported a 60% reduction in the volume of the regenerated tissues. On the other hand, while most stem cells are tooth-derived, studies on the impact of tooth maturation on the differentiation capacity of the stem cells has demonstrated that there is a reduced odontogenic, but enhanced osteogenic differentiation capacity the more mature the source (Lei et al. 2011). Finally, laser therapy has biostimulating properties that can assist proliferation of stem cells. Arany et al. (2014) investigated photo-modulation approaches. They reported mineralization and stimulation of stem cells due to the paracrine effect of activated factors and the large area of radiation (Arany et al. 2014).

The isolation approach for harvesting stem cells can affect their differentiation. In the study of Jeon et al. (2014) on two isolation approaches, an outgrowth method and enzymatic disaggregation, outgrowth SHEDs were more likely to differentiate into hard tissue forming cells while enzymatic disaggregated SHEDs were associated with more colony forming cells, adipogenic differentiation and overall stemness (Jeon et al. 2014).

### Time points

Most of the studies evaluated dentine-pulp regeneration from 4 to 8 weeks after transplantation (Tables 2, 3, 4 and 5). Of the 60 studies, 15 evaluated regeneration after 4 weeks and 16 evaluated regeneration after 8 weeks. Long-term evaluation (more than 20 weeks) was rare and limited to 6 studies (Tables 2, 3, 4 and 5). Those studies that used ectopic models of dentin-pulp complex evaluated the regeneration of the dentin-pulp from 2 to 8 weeks with the most frequent time point being after 8 weeks. Those studies that used true root canal models evaluated the regeneration of the new dentin-pulp complex from 2 to 26 weeks after surgery with the most frequent time point being 2 weeks (four studies) or the range of 2–4 weeks (Table 5). Apparently, some studies had multiple time points for their evaluation (Tables 2, 3, 4 and 5).

### Assessments

Before cell transplantation, immunocytochemistry, MTT assay, SEM and flowcytometric analyzes are routinely performed to characterize the transplanting stem cells. To evaluate the regenerating dentine-pulp complex, histology and histomorphometry, immunohistochemistry, and radiology (CT, micro-CT and plain radiography) are the gold standard methods. Histologic slides help to compare the amount of vascularization with the ratio of vessel surface to the entire surface of the slide (Zhang et al. 2015). Of 60 studies evaluated, all used histologic assessments, 36 used immunohistochemistry, 6 used immunofluroscent, 3 used immunostaining, 3 used micro-CT-scan, 4 used radiography, 4 used SEM and 1 study used MRI to evaluate pulp tissue regeneration (Tables 2, 3, 4 and 5). Immunohistochemistry was much more popular in retrograde studies as 31 from 40 studies with into renal capsule transplantation and subcutaneous transplantation used this method for their assessments (Tables 2 and 3). Radiography was not used in any retrograde studies.

## Discussion

To regenerate a necrotic pulp, just as with other tissues, three main components are needed. Vital cells in the root canal that can differentiate into the natural pulp cells, morphogenic factors to initiate and promote cell differentiation and a matrix that mechanically support the cells and provide an environment to sustain their vitality and proliferation (Galler et al. 2011).

In recent studies, various types of stem cells from various sources in the body have been manipulated for dentine-pulp regeneration (Tables 2, 3, 4 and 5). Dental pulp stem cells are the cell of choice in most of the studies and their capacity for regeneration of the dentine-pulp complex has been demonstrated (Tables 1, 2, 3, 4 and 5). Despite a greater tendency for regeneration of the dentine-pulp complex, administration of SCAP and SHED was rare (all studies that manipulated SCAP and SHED in Tabled 2, 3, 4 and 5 confirm this). Beside dental sources, stem cells from non-dental source such as bone marrow derived mesenchymal stem cells and adipose-derived stem cells were also able to regenerate pulp tissue (Murakami et al. 2015). Generally, each type of adult stem cell seems to be capable of dentine-pulp complex regeneration so that the selected stem cell should be the most feasible to use and the cheapest, especially when the main obstacle in guided tissue regeneration is the cost.

Third molars or any to-be-extracted tooth for orthodontic purposes, and not being extracted because of a microbial infection, are good sources of stem cells. The human body is a rich source of stem cells and they remain in their niches or circulate systemically around the body. In the presence of chemotactic gradients, these cells migrate to the site of injury and participate in the regeneration process, as their potential for migrating to the root canal has demonstrated (Ruangsawasdi et al. 2016; Zhang et al. 2015). In addition, transplanted stem cells may not remain effectively in the site of injury, but migrate elsewhere or go through apoptosis. Such events may be dependent on the type of stem cells, how prone they are to apoptosis, and the structure of the scaffold, which will impact upon the viability of the environment and its influence on stem cells migration. The optimum number of stem cells to be transplanted should be estimated experimentally for each type of stem cell and specific scaffold. However, systemic stem cells can take a role as a backup source. Factors such as SDF-1, SCF and G-CSF help to summon stem cells, and BMSCs particularly are used to demonstrate this in the root canal system (Ruangsawasdi et al. 2016; Takeuchi et al. 2015; Zhang et al. 2015).

Stem cells normally will not differentiate or if they do, they can differentiate to any type of cell. Therefore, their differentiation should be controlled by the means of appropriate growth factors. Soluble proteins of the dentine matrix provide a suitable environment for differentiation of stem cells into odontoblast-like cells while these proteins appear to control the natural differentiation of reparative stem cells. In addition, their position in their locality within dentine, peripheral to the pulp area could help the creation of an odontoblast-like cell lining integrated into the dentine wall. Third molars or any to-be-extracted teeth for orthodontic purposes are good source of autogenous dentine matrix. Yet, these matrixes are supposed to be preserved until needed. Freezing makes long-term preservation of human dentine matrix possible as it maintains the mechanical properties of the matrix (Chen et al. 2015b; Jiao et al. 2014).

Regeneration of the dentine-pulp complex relies on sufficient vascularization, which may be limited in the restricted apical region of a canal. Administering growth factors such as VEGF promotes vascularization, but it has a short half-life, so its systemic administration is limited (Li et al. 2016). Binding to heparin is a strategy to make VEGF bioavailable for longer (Li et al. 2016). Apart from locally administering such growth factors, treating stem cells under hypoxic conditions induces cells to secret vascularizing agents (Kuang et al. 2016). Presumably, when cells are in a deficient environment, they would secret a complex of growth factors to overcome the challenge. This type of cell can be used for regeneration purposes and lowers the costs of using purified growth factors.

The scaffolds should have the characteristics to aid in the regeneration of specific tissues. It should have controlled biodegradability to mechanically support the transplants, but not compete spatially with the regenerated tissue. Such controlled biodegradability will be created by a combination of long-term and short-term degradable materials. The proper proportion of this combination should be evaluated experimentally. The scaffold should be porous and spongy to be able to carry sufficient stem cells and growth factors and allow the stream of extracellular matrix and the formation of new blood vessels. As a carrier of growth factors, it should provide controlled release otherwise they will degrade rapidly and thus not take part in long-term regeneration. Binding to heparin can provide this slow release. Silk fibroin scaffold have been used widely in regeneration of tissues such as skin, bone, cartilage, etc., and its efficacy for pulp regeneration has been studied in vivo (Yang et al. 2015a). Treated dentine matrix, other than carrying dentinogenic growth factors, is also applicable as a scaffold (Chen et al. 2015a; Yang et al. 2012). Animal model studies on regeneration of dentine-pulp complex are performed mainly by transplantation of a complex of stem cell, growth factors and scaffolds (C-SGS) into the pulpectomized root canal, which is susceptible to various irritants such as bacteria, masticatory forces, restricted nutritional supply ,etc. Therefore, for final approval of novel treatment concepts, intracanal transplantation is necessary. However, as proof of principles for novel studies, C-SGS may be initially transplanted with or without a slice of a tooth subcutaneously or into the renal capsule to evaluate the potential of the novel complex for true regeneration of the dentine-pulp complex regardless of the side effects. This will help track the causes for potential failures and reduce costs in cases where the novel C-SGS proves to be inappropriate.

## Conclusion

It is important to realize that endodontic treatment of teeth with necrotic pulp using stem cells and suitable biomaterials results in pulp regeneration. However, feasibility of stem cell transplantation to treatment sites along with its cost may be obstacles for clinical use of such methods. Scaffolds and biomaterials provide a meaningful approach to better incorporate stem cells and growth factors along with controlled rate of regeneration. Therefore, we recommend future studies to focus on providing a clear guideline for suitable and preferable properties of biomaterials to be used in regenerative endodontics.
